# Left ventricular depression and pulmonary edema in rats after short-term normobaric hypoxia: effects of adrenergic blockade and reduced fluid load

**DOI:** 10.1007/s00424-021-02618-y

**Published:** 2021-09-12

**Authors:** Peter Appelt, Philipp Gabriel, Christian Bölter, Nicole Fiedler, Katrin Schierle, Aida Salameh, Beate Rassler

**Affiliations:** 1 Carl-Ludwig-Institute of Physiology, University of Leipzig, Leipzig, Germany; 2 Institute of Pathology, University of Leipzig, Leipzig, Germany; 3 Department of Pediatric Cardiology, Heart Centre, University of Leipzig, Leipzig, Germany

**Keywords:** Normobaric hypoxia, Adrenergic blockade, Fluid load, Cardiac function, Pulmonary edema, Inflammation

## Abstract

Acute normobaric hypoxia may induce pulmonary injury with edema (PE) and inflammation. Hypoxia is accompanied by sympathetic activation. As both acute hypoxia and high plasma catecholamine levels may elicit PE, we had originally expected that adrenergic blockade may attenuate the severity of hypoxic pulmonary injury. In particular, we investigated whether administration of drugs with reduced fluid load would be beneficial with respect to both cardiocirculatory and pulmonary functions in acute hypoxia. Rats were exposed to normobaric hypoxia (10% O_2_) over 1.5 or 6 h and received 0.9% NaCl or adrenergic blockers either as infusion (1 ml/h, increased fluid load) or injection (0.5 ml, reduced fluid load). Control animals were kept in normoxia and received infusions or injections of 0.9% NaCl. After 6 h of hypoxia, LV inotropic function was maintained with NaCl injection but decreased significantly with NaCl infusion. Adrenergic blockade induced a similar LV depression when fluid load was low, but did not further deteriorate LV depression after 6 h of infusion. Reduced fluid load also attenuated pulmonary injury after 6 h of hypoxia. This might be due to an effective fluid drainage into the pleural space. Adrenergic blockade could not prevent PE. In general, increased fluid load and impaired LV inotropic function promote the development of PE in acute hypoxia. The main physiologic conclusion from this study is that fluid reduction under hypoxic conditions has a protective effect on cardiopulmonary function. Consequently, appropriate fluid management has particular importance to subjects in hypoxic conditions.

## Introduction

Exposure to acute hypoxia may induce pulmonary injury characterized by pulmonary edema (PE). This is a typical complication in travelers to high altitude called high-altitude PE (HAPE). Although hypoxia-induced PE such as HAPE is considered a hydrostatic edema [4, 29], it is accompanied by upregulation of pro-inflammatory cytokines and inflammation in the lung [21, 28].

Hypoxia is associated with increased sympathetic activity [23, 30]. High plasma levels of catecholamines (CA) may also elicit pulmonary injury with PE and inflammation as demonstrated in experiments with intravenous (i.v.) CA administration [27, 36] or observed in clinical conditions such as pheochromocytoma [12, 48] or neurogenic PE [34, 42]. In rats, infusion of adrenergic agonists induced development of PE and a significant increase of IL-1 and IL-6 mRNA in the lung [37, 39]. These CA-induced pulmonary injuries showed great similarities with those induced by normobaric hypoxia [38]. Experimental and clinical studies demonstrated that pro-edematous and pro-inflammatory effects are predominantly exerted by α-adrenergic mechanisms [1, 11]; however, β-adrenergic pathways can contribute to this pathology as well [36, 43]. Sympathetic activation in hypoxia has been ascribed to stimulation of peripheral chemoreceptors [22, 23, 30]. Subjects susceptible to HAPE showed an exaggerated increase in sympathetic firing rate under hypoxic conditions, which preceded development of PE [16]. The similar patterns of lung injury in the two conditions suggest that hypoxia-induced PE and inflammation might be due, at least in parts, to sympathetic activation. Therefore, we had expected that adrenergic blockade would attenuate the severity of hypoxic PE and inflammation.

A previous study on rats [6] showed that normobaric hypoxia induced a depression of left ventricular (LV) systolic pressure (LVSP) that appeared almost simultaneously with PE (after 6 h of hypoxia). Studies on animals under hypoxic conditions showed a decrease in LV contractility associated with a compromised LV energy metabolism [41, 45]. In particular, mitochondrial respiration and ATP synthesis are impaired in hypoxic LV myocytes [3, 19]. Hypoxia plus infusion of norepinephrine as a model of sympathetic overstimulation in hypoxia delayed LVSP depression and aggravated PE. Hypoxia plus adrenergic blockade deteriorated hypoxic LVSP depression but did not prevent formation of PE. These results suggested that hypoxic LVSP depression might have counteracted the supposed mitigating effects of adrenergic blockers on PE [6]. Physiologic responses to acute exposure to hypoxia include a reduction in plasma volume due to increased diuresis [17, 18]. In the previous rat study [6], the drugs were administered as an infusion at a rate of 1 ml/h. Given an average blood volume in young female SD rats of 7.8 ml/100 g body weight [33], this means a fluid input of about 6% per hour related to total blood volume. This corresponds to a more than threefold infusion rate as administered to non-hypovolemic patients in clinical settings. We assume that drug administration to the hypoxic rats by infusion might compromise their fluid management, in particular, when infusion is given over several hours. This would entail additional load to the LV causing backlog into the pulmonary circulation and into the right ventricle (RV) and, hence, aggravating LV dysfunction and impeding attenuation of PE.

Therefore, we designed the present study to investigate some questions related to the importance of additional fluid load and sympathetic activity for cardiopulmonary function under conditions of acute hypoxia. We compared increased fluid load (infusion at 1 ml/h) with reduced fluid load achieved by a single injection with low fluid volume prior to the exposure to hypoxia. The role of fluid load is studied with and without adrenergic blockade. Adrenergic blockade serves as a model of suppression of hypoxia-induced sympathetic effects. To the best of our knowledge, this is the first study directly comparing effects of low versus high fluid load and drug administration in acute hypoxia. Specifically, we investigated whether (a) increased fluid load aggravates cardiocirculatory dysfunction and hypoxia-induced pulmonary injury and (b) adrenergic blockade may prevent or attenuate pulmonary injury when additional fluid load is low.

## Materials and methods

### Animal model

All experiments were performed on 147 female Sprague–Dawley rats supplied by Charles River (Sulzfeld, Germany). The body weight was 225 ± 23 g at the beginning of the study corresponding to an age of about 12 weeks. All animal protocols were approved by the Federal State Agency. The experiments were conducted in accordance with the Guide for the Care and Use of Laboratory Animals published by the National Institutes of Health and with the “European Convention for the Protection of Vertebrate Animals used for Experimental and other Scientific Purposes” (Council of Europe No 123, Strasbourg 1986).

### Study protocol

Animals were exposed to normoxia (N) or normobaric hypoxia (H) for 1.5 or 6 h. For exposure to hypoxia, the animals were placed into a hypoxic chamber sized 65 × 105 × 50 cm. The gas mixture in the chamber contained 10% oxygen in nitrogen. Special equipment prevented penetration of ambient air during manipulations on the animals, thus keeping the oxygen concentration in the chamber stable at 10 ± 0.5%. The hypoxia-exposed animals were treated with adrenergic blockers or with 0.9% sodium chloride (NaCl) solution. We used the α-adrenergic blocker prazosin (PZ), the β-adrenergic blocker propranolol (PR), or a combination of both (PZ + PR; for doses see Table 1). Normoxic control animals remained under room air condition and received NaCl solution.

**Table 1:** List of treatments

Group		Amount of fluid (number of animals)	
Treatment: drug dose		Infusion (inf)	Injection (inj)
Normoxic control: (NaCl,N)			
1.5 h		1.5 ml (*n* = 8)	0.5 ml (*n* = 6)
6 h		6 ml (n = 9)	0.5 ml (*n* = 6)
Hypoxic control: (NaCl,H)			
1.5 h		1.5 ml (*n* = 9)	0.5 ml (*n* = 5)
6 h		6 ml (*n* = 10)	0.5 ml (*n* = 6)
Hypoxia + adrenergic blockade			
** α**: Prazosin: 0.1 mg kg^−1^ h^−1^ (PZ,H)			
1.5 h:	0.15 mg kg^−1^	1.5 ml (*n* = 9)	0.5 ml (*n* = 9)
6 h	0.6 mg kg^−1^	6 ml (*n* = 8)	0.5 ml (*n* = 5)
** β**: Propranolol: 1 mg kg^−1^ h^−1^ (PR,H)			
1.5 h:	1.5 mg kg^−1^	1.5 ml (*n* = 6)	0.5 ml (*n* = 9)
6 h	6 mg kg^−1^	6 ml (*n* = 7)	0.5 ml (*n* = 7)
** α** + **β**: PZ: 0.1 mg kg^−1^ h^−1^ + PR 1 mg kg^−1^ h^−1^ (PZ + PR,H)			
1.5 h:	PZ 0.15 + PR 1.5 mg kg^−1^	1.5 ml (*n* = 7)	0.5 ml (*n* = 6)
6 h	PZ 0.6 + PR 6 mg kg^−1^	6 ml (*n* = 6)	0.5 ml (*n* = 3)*
6 h	PZ 0.6 + PR 3 mg kg^−1^		0.5 ml (*n* = 6)

^*^These animals died immediately after injection and were therefore excluded from evaluation

The fluid was administered either by i.v. injection at the start of the experiment (inj) or i.v. infusion over the total experimental time (inf). The infusion rate was 1 ml/h, while the amount of injection fluid was 0.5 ml. The drug doses were chosen according to previous experiments [36]. They were equal for corresponding inf and inj groups, but the fluid volume was less for the inj groups (see Table 1). Only for the group 6 h PZ + PR,H-inj, we had to reduce the PR dose as the first animals died immediately after injection from acute cardiac failure. These animals were discarded for further analyses. For the final experiments, we chose the maximal PR dose to be survived, which was at 0.5 mg PR kg^−1^ h^−1^. Except for PZ (Pfizer, Karlsruhe, Germany), all drugs were obtained from Sigma-Aldrich (Deisenhofen, Germany).

For fluid application, the animals were anesthetized, and the left jugular vein was prepared. Injection was given within 15 s into the vein, which was ligated afterwards. For infusion, a catheter (Vygon, Aachen, Germany) was inserted into the vein, and the fluid was administered with an automatic pump (Infors AG, Basel, Switzerland). For experiments over 6 h, this operation was performed in 2% isoflurane anesthesia. These animals woke up after injection or catheter insertion and moved freely with access to tap water and rat chow diet (Altromin C100, Altromin GmbH, Lage, Germany). Animals allocated to 1.5 h of treatment were anesthetized with an intraperitoneal (i.p.) injection of thiopental sodium (Trapanal®, Byk Gulden, Konstanz, Germany) 80 mg kg^−1^ and remained in anesthesia until the end of the experiment. Exposure to hypoxic environment started immediately after this initial operation.

### Hemodynamic measurements

Hemodynamic measurements were performed in anesthesia with thiopental sodium (Trapanal®, 80 mg kg^−1^, i.p.) and started about 30–40 min before the end of the exposure time. The animals were tracheotomized, and a polyethylene cannula was placed in the trachea. RV and LV were catheterized with Millar® (Millar Instruments, Houston, TX) ultraminiature catheter pressure transducers (for more details see [35]) to measure heart rate (HR), RV, and LV systolic pressures (RVSP, LVSP). In addition, RV and LV maximal rise (dP/dt max) and maximal drop in systolic pressure (dP/dt min) were determined as measures of ventricular contractility and relaxation, respectively. After withdrawal of the LV catheter tip into the aorta, diastolic aortic pressure (DAP) was measured to calculate mean aortic pressure (MAP). Cardiac index (CI, body mass-related cardiac output) was determined by thermodilution using a thermosensitive 1.5F microprobe and a Cardiomax II computer (Columbus Instruments, Columbus, OH). The total peripheral resistance (TPR) was calculated by dividing MAP by CI. Hypoxic animals remained in hypoxia until completion of hemodynamic measurements.

### Sampling of materials

Animals were sacrificed by exsanguination from the abdominal aorta. In the next step, we opened the thoracic wall and collected pleural fluid (PF). After ligation of the right main bronchus, a bronchoalveolar lavage (BAL) was performed two times consecutively with 3 ml 0.9% NaCl each. The fluid was instilled via the tracheal cannula into the left lung and withdrawn immediately. The recovery rate was about 90% on average. Heart and right lung were then rapidly excised. Tissue samples of the cardiac apex and of the upper and lower lobes of the right lung were fixated in formalin for histological analysis. The blood was centrifuged for 10 min at 2100 rpm. Serum, BAL fluid, and further samples of lung tissue were frozen and stored at -80 °C for further analyses.

### Lung histology, wet-to-dry weight ratio, and BAL cytology

The formalin-fixated lung tissue samples were embedded in paraffin, sliced, and stained with hematoxylin–eosin (HE). Histological assessments were done by two independent investigators (PG and KS), who were blinded towards the treatment group. In the lungs, they evaluated pulmonary edema (PE), inflammation, and vascular hypertrophy. For a detailed quantification of PE, about 16–24 single slices from each animal (8–12 slices each from the upper and lower lung lobe) were evaluated. First, PE severity in each area of the section was scored with 0 (absent), 1 (mild), 2 (moderate), and 3 (severe). As no differences between upper and lower lung lobes were apparent, values from all sections of the two lobes were pooled. The pulmonary edema index (PEI) was calculated by cumulating the products of edema score and proportionate area of each part of all slices [6].

For determination of lung wet-to-dry weight ratio (W/D ratio), lung tissue samples were weighed immediately after preparation (wet weight, W) and after drying in an oven at 75 °C for 48 h (dry weight, D). The W/D ratio served as a surrogate parameter of water accumulation in the lung.

BAL fluid was centrifuged for 20 min at 1500 rpm in a cytocentrifuge. The cytologic preparation was stained with HE and evaluated by a pathologist (KS) who was not aware of the treatment of the animal. The number of macrophages, neutrophils, lymphocytes, and eosinophils was given in percent of the total cell number.

### RNA isolation and polymerase chain reaction (PCR) analysis

#### RNA isolation

Rat total RNA was isolated from lung tissue by lysing homogenization with the TRIzol® reagent (Gibco BRL, Karlsruhe, Germany), followed by Direct-zol RNA miniprep according to the manufacturer’s instructions (Direct-zol RNA Miniprep Kit, Zymo Research, Freiburg, Germany). The quality and quantity of RNA were determined with a NanoDrop™ 2000 spectrophotometer (Thermo Scientific, Waltham, MA, USA).

#### Real-time PCR (RT-PCR) analysis

Two micrograms of total RNA were transcribed into cDNA using the Transcriptor First Strand cDNA Synthesis Kit (Roche Applied Science, Mannheim, Germany). Approximately 10% of the cDNA was applied for measuring the amount of the target genes as defined in Table 2. As inflammatory markers, we determined the expression of interleukin (IL)-6 and tumor necrosis factor α-induced protein 1 (TNFAIP1) in the lung. Short fluorescein amidite (FAM)-labeled hydrolysis probes (Universal ProbeLibrary (UPL), Roche Diagnostics GmbH, Mannheim, Germany) were used for RT-PCR reactions. Primers and the UPL probe were designed by ProbeFinder version 2.50 for rat (Roche Applied Science, Mannheim, Germany).

**Table 2:** List of primers used for RT-PCR

Gene	AN	UPL number	f/r	Primer sequence	Position
IL-6	#106	NM_012589.1	f	cct gga gtt tgt gaa gaa caa ct	433–455
			r	gga agt tgg ggt agg aag ga	555–574
TNFAIP1	#81	NM_182950.4	f	cca ctt ggc tga gag gaa ag	136–155
			r	caa atg agt gtc ccg cag a	213–195
ARPP	#18	NM_031660.1	f	ttt aag gaa aag att gca gaa agg	213–195
			r	cac cag tga cct ctg tct tat cc	268–246

*AN* accession number, *f* forward, *r* reverse, *IL-6* interleukin 6, *TNFAIP1* tumor necrosis factor α-induced protein 1, *ARPP* cyclic AMP-regulated phosphoprotein-19

The amount of the target genes was determined using semiquantitative RT-PCR with a Light Cycler® 480 System (Roche Diagnostics GmbH, Mannheim, Germany) according to the manufacturer’s instructions with a pre-incubation step at 95 °C for 5 min followed by 45 cycles at 95 °C for 10 s, 60 °C for 15 s, and 72 °C for 4 s. The relative amount of the target genes was calculated by the threshold cycle (Ct) and the 2-ddCt method (Software Version LCS480 1.5 0.39, Roche Diagnostics GmbH, Mannheim, Germany) using cyclic AMP-regulated phosphoprotein-19 (ARPP) as the housekeeping gene. The expression of specific mRNAs has been normalized to ARPP mRNA expression.

### Catecholamine concentration in serum

Serum concentrations of epinephrine (Epi) and norepinephrine (NE) were measured by high-pressure liquid chromatography (HPLC) using a commercial HPLC assay (Chromsystems, Martinsried, Germany) based on the method of Bauersfeld [5]. Sample processing and performance of the HPLC were carried out according to the manufacturer’s instructions. Catecholamines were detected using an electrochemical detector EC3000 (Recipe, Munich, Germany).

### Statistical analysis

Data are presented as means ± SEM. Statistical analyses were carried out with the R version 4.0.3 (R project for Statistical Computing, Vienna Austria). We performed separate ANOVAs for comparing (a) NaCl,N versus NaCl,H groups and (b) NaCl,H groups versus all hypoxic groups with adrenergic blockers. Post hoc analyses were performed using Fisher’s least significant difference (LSD) test. In addition, we calculated Cohen’s *f* as a measure of effect size with *f* values of 0.10, 0.25, and 0.40 indicating low, medium, and large effects, respectively [10]. To compare serum CA concentrations of normoxic and hypoxic animals, a Mann–Whitney rank sum test was used. The effect size was assessed using Cohen’s *d* for two independent groups with *d* values of 0.20, 0.50, and 0.80 indicating low, medium, and large effects, respectively [10].

## Results

### Hemodynamic function

**Fig. 1:**
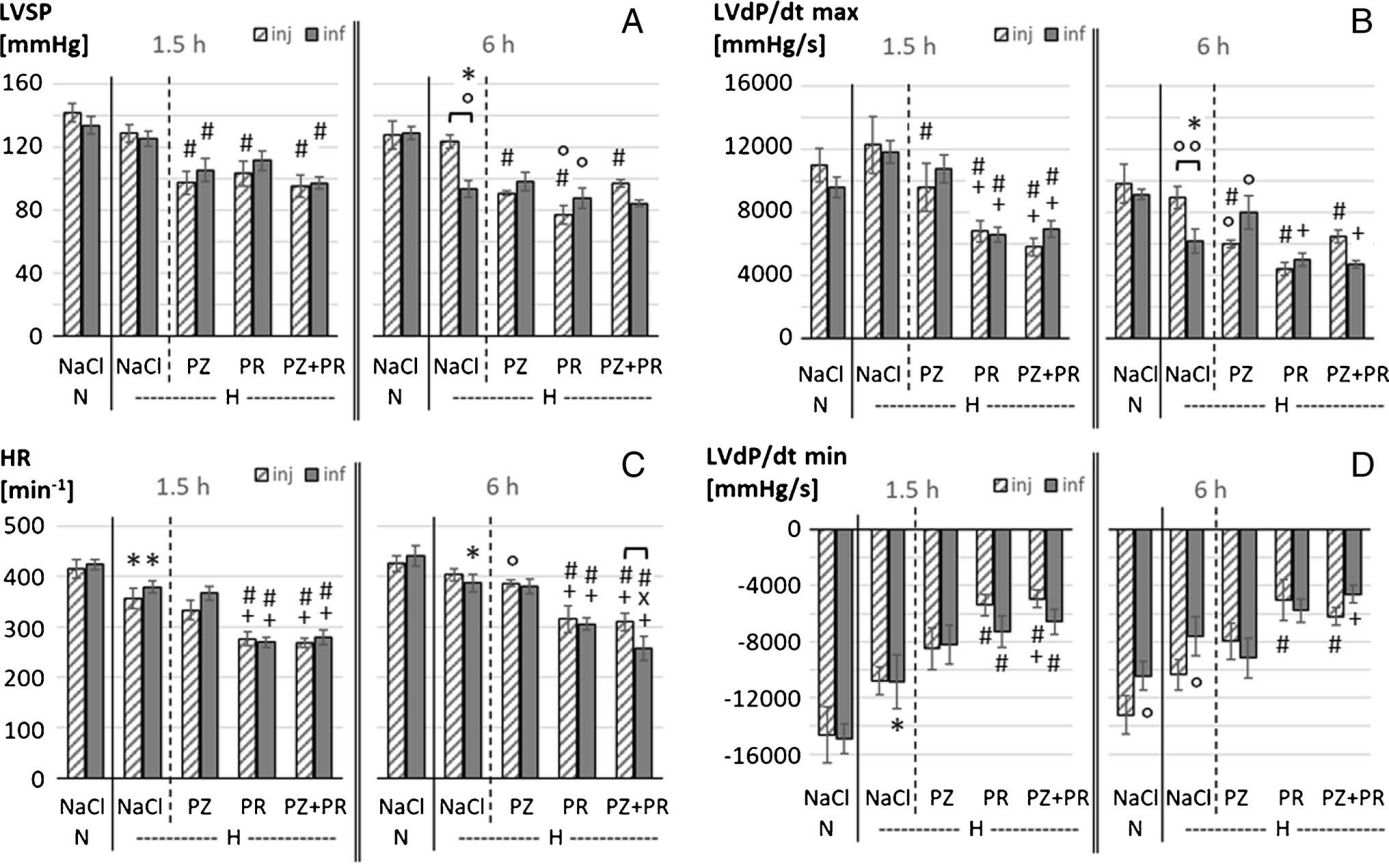
Hemodynamic function: **A** LVSP, left ventricular systolic pressure (mmHg); **B** LV dP/dt max, left ventricular contractility (mmHg/s); **C** HR, heart rate (min^−1^); and **D** LV dP/dt min, left ventricular relaxation (mmHg/s). In each panel, left parts show the results of 1.5-h experiments; right parts show the results of 6-h experiments; N, normoxia; H, hypoxia; PZ, prazosin; PR, propranolol. Data are given as means ± SEM. Hatched boxes represent injection groups and gray boxes infusion groups. Significant marks: * significant vs. corresponding NaCl,N group; # significant vs. corresponding NaCl,H group; + significant vs. corresponding PZ,H group; x significant vs. corresponding PR,H group; ° significant vs. corresponding 1.5 h group. ┏┓difference between related injection and infusion groups

Hemodynamic results are presented in Fig. 1 (LVSP, LV dP/dt max, LV dP/dt min, HR) and in Table 3 (MAP, DAP, TPR, CI, RVSP, RV dP/dt max, RV dP/dt min).

**Table 3:**
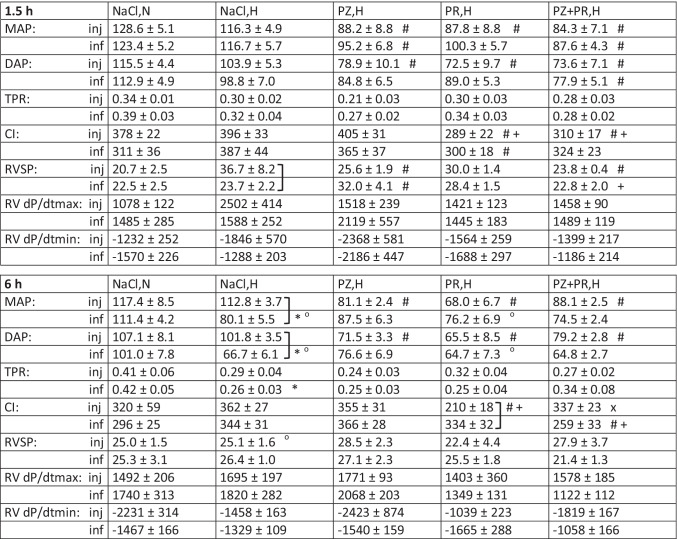
Hemodynamic data

Values are given as means ± SEM. *MAP* mean arterial pressure (mmHg), *DAP* diastolic aortic pressure (mmHg), *TPR* total peripheral resistance (mmHg min kg ml^−1^), *CI* cardiac index (ml min^−1^ kg^−1^), *RVSP* right ventricular systolic pressure (mmHg), *RV dPdt max/min* right ventricular maximal rise/maximal drop in systolic pressure (mmHg s^−1^). Significant marks: * NaCl,H significant vs. related NaCl,N; ° 6 h group significant vs. related 1.5 h group;] significant between corresponding inj and inf groups; hypoxia and adrenergic blockers: # significant vs. related NaCl,H; + significant vs. related PZ,H; x significant vs. related PR,H

### Effect of acute hypoxia (hypoxic vs. normoxic controls)

Acute hypoxia induced a depression of LV function. After 1.5 h of hypoxia, LVSP decreased mildly, while HR was significantly reduced (*p* = 0.018, *f* = 0.63). These effects were similar with infusion and injection. LV relaxation (LV dP/dt min) also decreased, and this was significant with NaCl infusion (*p* = 0.007, *f* = 0.73). In contrast, LV contractility (LV dP/dt max) was slightly improved.

After 6 h of hypoxia, LVSP and LV dP/dt max decreased significantly compared to normoxic controls (*p* < 0.001, *f* = 1.15 and 0.93, respectively), but only with infusion. The reduction of LVSP was associated with a significant reduction in MAP and DAP (*p* < 0.001, *f* = 1.26 and 1.04, respectively, see Table 3). These depressor effects were significantly weaker or even completely absent with injection. Similarly, LV dP/dt min decreased compared to 1.5 h of hypoxia with infusion but not with injection. HR did not further decrease with infusion, but rather showed slight recovery with injection.

TPR also decreased after 6 h of hypoxia, particularly with infusion (*p* = 0.028, *f* = 0.7). Correspondingly, CI was even slightly elevated compared to normoxic controls, and this occurred both with infusion and injection. RV function was not impaired by hypoxia. Only in the 1.5 h NaCl,H-inj group, RVSP was significantly higher than in all other hypoxic control groups (*p* = 0.002, *f* = 0.54, see Table 3).

### Additional effect of adrenergic blockade in acute hypoxia

Short hypoxia exposure (1.5 h) without adrenergic blockade only mildly affected LV inotropic function. Administration of adrenergic blockers, however, exerted a strong depressive effect on LVSP and LV dP/dt max compared to hypoxic controls (*p* < 0.001, *f* = 1.03 and 1.21, respectively). This was similar for 6 h of hypoxia with reduced fluid application (inj). In contrast, while 6 h of hypoxia with NaCl infusion had significantly depressed LVSP and LV dP/dt max, infusion of adrenergic blockers had no further significant effect on these parameters. Similar results were obtained for MAP and DAP (see Table 3). With β-adrenergic or dual adrenergic blockade, most of the LV effects were significantly stronger than with α-adrenergic blockade; in particular, there was a decrease of CI (*p* = 0.002, *f* = 0.77). In addition, β-blockade (alone or in combination with PZ) exerted significant negative chronotropic effects compared to corresponding hypoxic controls (*p* < 0.001, *f* = 1.31). No significant effects were exerted by adrenergic blockade on RV function and on TPR compared to hypoxic controls.

### Lung

#### Pulmonary edema and pleural fluid

**Fig. 2:**
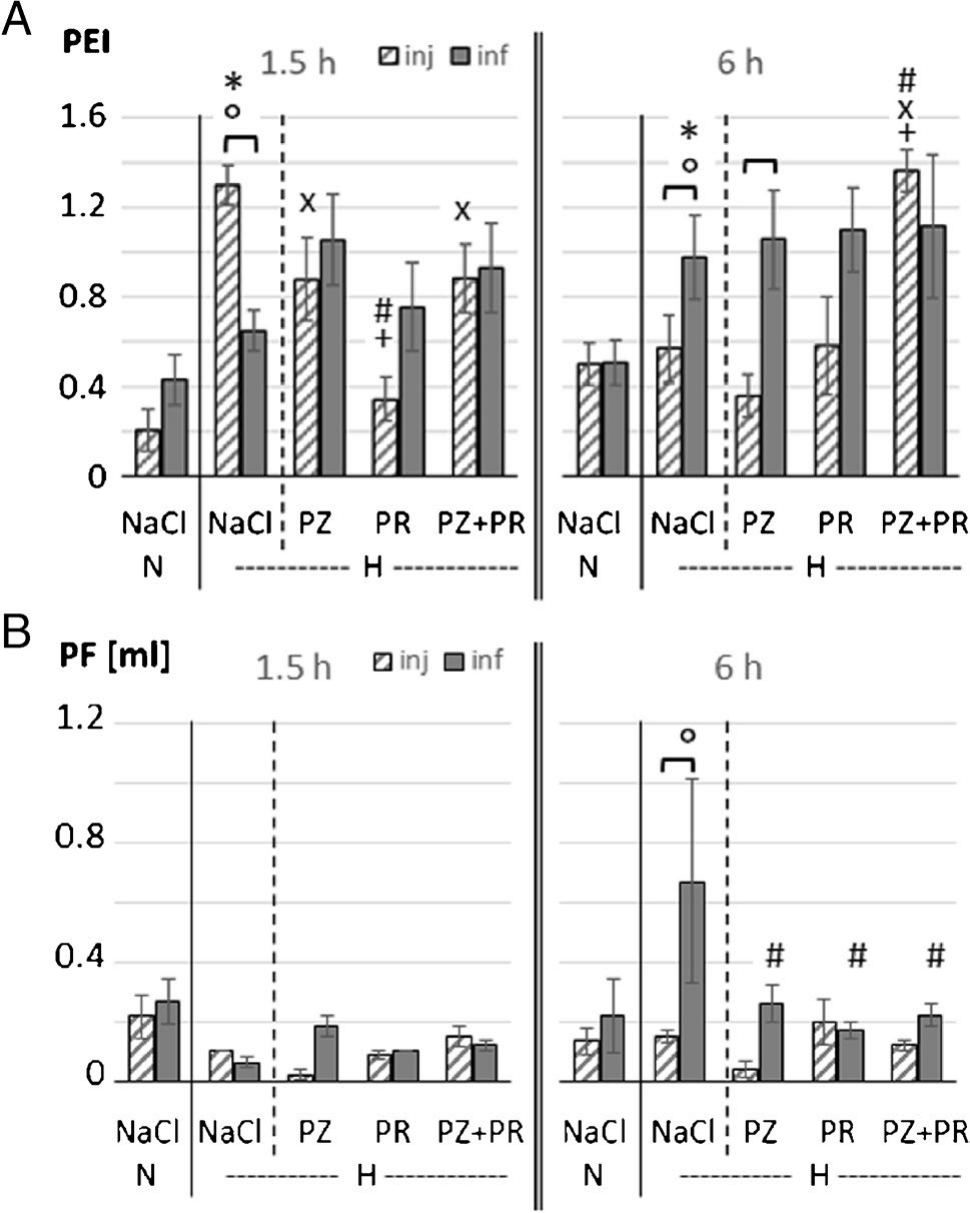
**A** PEI, pulmonary edema index and **B** pleural fluid volume (ml). In each panel, left parts show the results of 1.5-h experiments; right parts show the results of 6-h experiments; N, normoxia; H, hypoxia; PZ, prazosin; PR, propranolol. Data are given as means ± SEM. Hatched boxes represent injection groups and gray boxes infusion groups. Significant marks: * significant vs. corresponding NaCl,N group; # significant vs. corresponding NaCl,H group; + significant vs. corresponding PZ,H group; x significant vs. corresponding PR,H group; ° significant vs. corresponding 1.5 h group. ┏┓difference between related injection and infusion groups

**Fig. 3:**
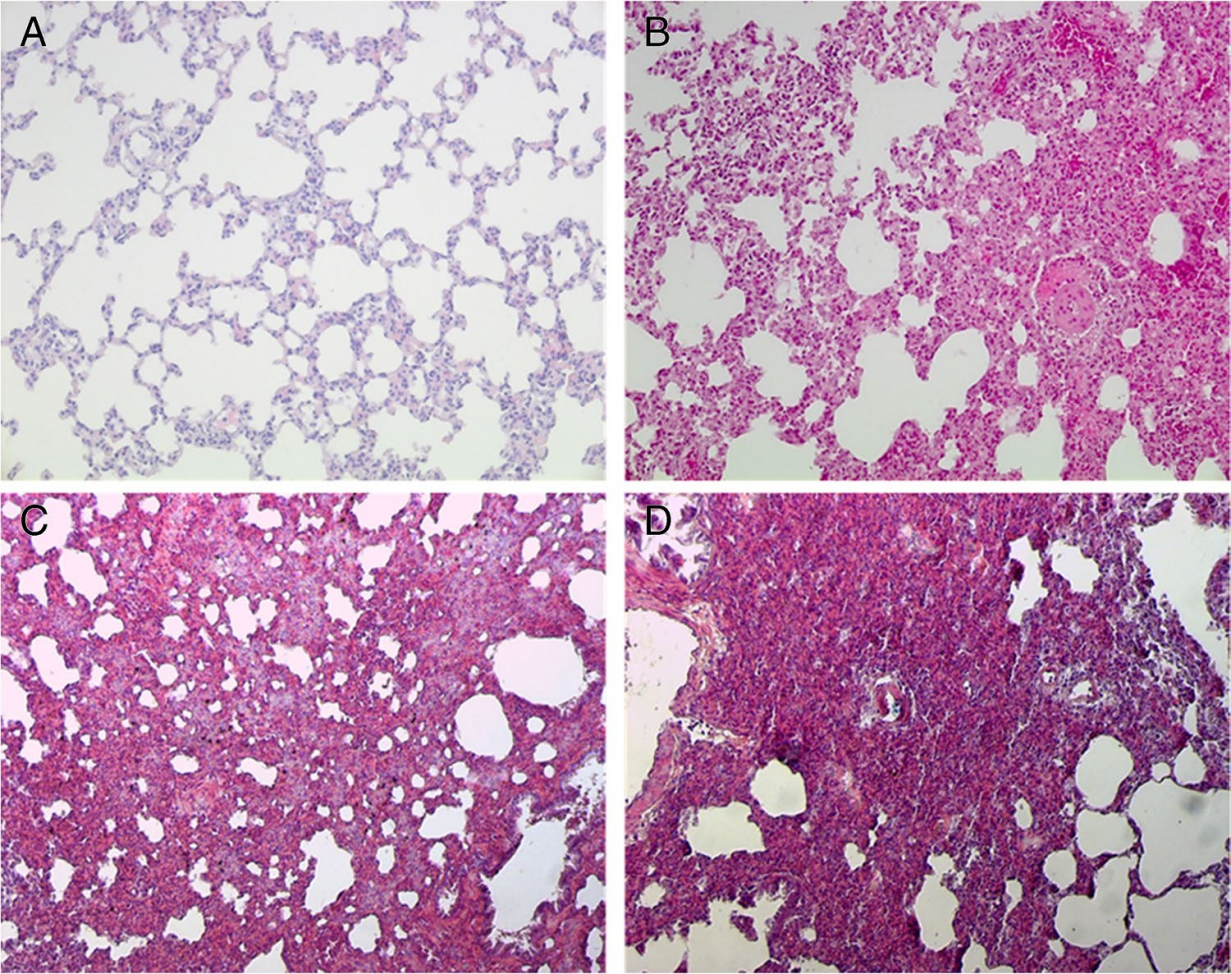
Lung histology: **A** 1.5 h NaCl,N-inj, no edema; **B** 1.5 h NaCl,H-inj, moderate edema; **C** 6 h PR,H-inf, moderate to severe edema; and **D** 6 h PZ + PR,H-inj, severe edema. All slices: original magnification × 20

In all normoxic controls, there were no or only mild histologic signs of PE. Acute hypoxia significantly aggravated PE with Cohen’s *f* indicating a strong effect (*f* = 0.88). In the 1.5 h NaCl,H-inj group, PEI was with 1.3 ± 0.09 more than sixfold of the 1.5 h NaCl,N-inj group and about twice as high as in the corresponding infusion group (*p* < 0.001; Fig. 2A and Fig. 3B). In the 6 h hypoxic control groups, PEI was significantly higher with infusion than with injection (*p* < 0.031). Contrasting to what we had expected, hypoxia and adrenergic blockade did not significantly attenuate PE severity but rather increased PEI, even with fluid injection. The differences between the hypoxic groups were large (*f* = 0.67). In general, PEI was higher with infusion than with injection with the exception of the 1.5 h NaCl,H-inj and the 6 h PZ + PR,H-inj groups (Fig. 2A, Fig. 3B and D). Of note, with the original dose of PR, the 6 h PZ + PR,H-inj rats died immediately after injection. To ensure the survival of these animals, the PR dose was reduced by half. Despite the reduced PR dose, PEI was even higher than in the related PR,H-inj group (*p* = 0.006).

Lung histology showed that PE occurred in patches that were spread over the total lung. PE was predominantly confined to the interstitium with alveoli mostly remaining free from fluid (Fig. 3). This was similar in all groups and was independent of infusion or injection. Correspondingly, the W/D ratio ranged between 4.8 and 5.4 (through all groups) confirming interstitial PE without severe alveolar affection (data not shown).

Formation of PE is often counteracted by fluid filtration into the pleural space as an important drainage pathway. In the 6 h NaCl,H-inf group, PF volume was significantly higher than in all other hypoxic control groups (*p* = 0.006, *f* = 0.65). Adrenergic blockers significantly decreased PF volume in the 6 h H-inf groups (Fig. 2B). In general, groups with higher PEI values such as groups with fluid infusion often developed higher PF amounts as a countermeasure. However, there were exceptions, e.g., in the 1.5 h NaCl,H-inj and 6 h PZ + PR,H-inj groups, which presented particularly high PEI values but very low PF volumes.

### Markers of inflammation

**Fig. 4:**
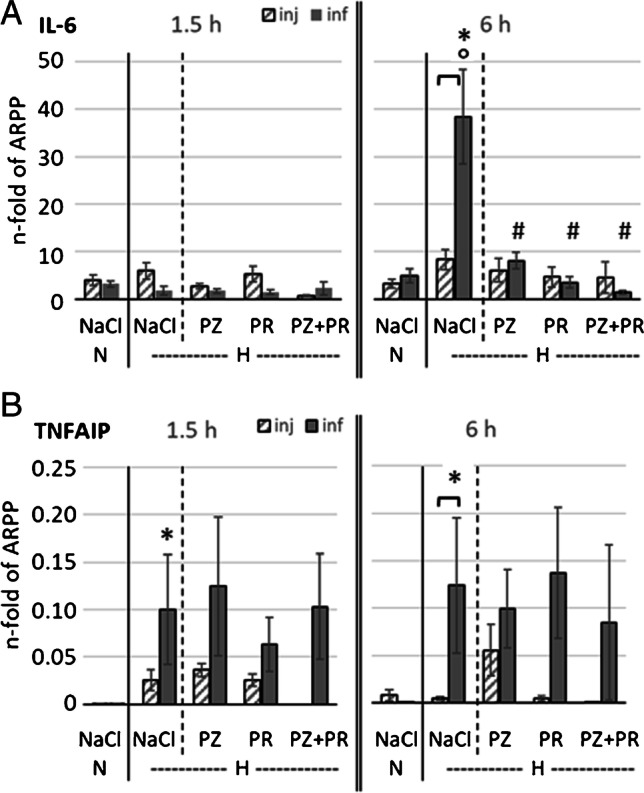
mRNA expression of inflammation markers in the lung:** A** IL-6, Interleukin-6 and **B** TNFAIP1, Tumor necrosis factor α-induced protein 1. Values are given as n-fold of ARPP mRNA expression. In each panel, left parts show the results of 1.5-h experiments; right parts show the results of 6-h experiments; N, normoxia; H, hypoxia; PZ, prazosin; PR, propranolol. Data are given as means ± SEM. Hatched boxes represent injection groups and gray boxes infusion groups. Significant marks: * significant vs. corresponding NaCl,N group; # significant vs. corresponding NaCl,H group; ° significant vs. corresponding 1.5 h group. ┏┓difference between related injection and infusion groups

About 28% of the hypoxic animals presented mild symptoms of inflammation in lung histology, and only 3% showed moderate inflammation signs; the others were free from visible inflammation. There were no clear differences between the hypoxic groups. No significant changes in the distribution of macrophages, neutrophils, and lymphocytes were found in BAL cytology (data not shown). The expression of the inflammatory markers IL-6 and TNFAIP1 increased under hypoxic conditions (*f* = 1.50 and 0.86, respectively), in particular when fluid was infused. IL-6 expression increased significantly compared to normoxic controls in the hypoxic control group after 6 h of fluid infusion but not after injection (*p* < 0.001). Adrenergic blockade completely abrogated this effect irrespective of application type (*f* = 1.47; Fig. 4A). We observed a moderate increase in TNFAIP1 mRNA after 1.5 h of hypoxia in animals with NaCl infusion but not with injection. With longer exposure to hypoxia (6 h) and NaCl infusion, TNFAIP1 expression increased significantly above the level in the time-corresponding NaCl,N-inf group (*p* = 0.005). Infusion of adrenergic blockers did not reduce it compared to hypoxic controls (Fig. 4B). In contrast, TNFAIP1 expression remained low after injection of adrenergic blockers (*f* = 0.64).

### Catecholamine concentration in serum

NE and Epi concentrations in the serum of normoxic animals were 2193 ± 298 and 4601 ± 603 pg/ml, respectively. In the hypoxic groups, NE and Epi concentrations varied widely. No significant differences among the groups were found. To compare differences between normoxic and hypoxic animals, we calculated the grand mean over all hypoxic animals. Although hypoxia increased CA concentrations to about five-fold of the normoxic values, Cohen’s *d* revealed this to be a rather small effect (NE: 12,125 ± 4313 pg/ml, *p* = 0.29, *d* = 0.27; Epi: 21,245 ± 8464 pg/ml, *p* = 0.045, *d* = 0.23).

## Discussion

### Effects of hypoxia, fluid load, and adrenergic blockade on cardiovascular function

The hemodynamic results of this study are in line with previous findings in animals under acute hypoxia [40]. In addition, they confirmed the results from the preceding study that acute hypoxia exerts direct negative inotropic effects on the LV myocardium [6]. However, in hypoxic control animals (6 h of hypoxia), only infusion but not injection induced a significant decrease in LVSP. LV contractility and relaxation (LV dP/dt max and LV dP/dt min) were also significantly reduced in the 6 h NaCl,H-inf group indicating an adverse effect of additional fluid load on LV inotropic and lusitropic functions under hypoxic conditions. These changes along with the decrease of HR suggest that the expected sympathetic activation was not able to fully compensate for the cardiodepressive hypoxic effects, at least with additional fluid load. The mild effects of hypoxia on serum NE and Epi concentrations suggest a rather weak hypoxic sympathetic activation, which is in line with our findings from a previous study in hypoxic rats [6]. Tachycardia is widely considered to be the typical cardiac response to hypoxia [30]. However, early studies showed that bradycardia is the primary response to peripheral chemoreceptor stimulation but is often masked by a secondary tachycardia resulting, at least in parts, from the ventilatory response to hypoxia [14]. In contrast, cardiac output was even slightly improved in hypoxia, and this is most probably due to a decrease in TPR. In the systemic vasculature, hypoxia induces vasodilation, which is mediated by non-nerval mechanisms including ATP-sensitive K^+^ channels, NO production, and effects of circulating Epi [13, 15].

Sympathetic activity contributes to preserve LV inotropic function under hypoxic conditions. With low fluid load (injection or infusion over 1.5 h), LV function was largely maintained when sympathetic effects were unrestricted. Application of adrenergic blockers, particularly β-adrenergic blockers, significantly decreased LVSP und LV dP/dtmax emphasizing the role of sympathetic activity in hypoxia. TPR remained on the low level of hypoxic controls. This response to hypoxia plus β-adrenergic blockade was completely different from that observed under normoxic conditions: In healthy normoxic Wistar-Kyoto rats, injection of propranolol did not significantly reduce or even slightly increased systolic pressure [20, 24]. Adrenergic blockade in normoxia primarily modulated TPR leading to secondary changes in arterial pressure [49], while in hypoxia, TPR was rather insensitive to adrenergic blockers.

The depression of LV inotropic and lusitropic functions after 6 h of hypoxia and fluid infusion, which was not further deteriorated by addition of adrenergic blockers, suggests that prolonged hypoxia combined with increased fluid load may reduce sympathetic activation or its cardiovascular effectivity. A possible explanation might be a reflex response to mechanical stimulation of cardiac ventricular receptors, which are activated, e.g., by blood volume expansion [44]. As a response, sympathetic activity is inhibited, while parasympathetic activity increases resulting in bradycardia and vasodilation. This reflex, known as Bezold-Jarisch reflex, can also be elicited in pathological situations such as myocardial ischemia by activation of both ventricular mechanoreceptors and chemoreceptors [2, 50]. Moreover, a reduced energy state of the hypoxic myocardium due to compromised mitochondrial respiration and ATP synthesis [3, 19] might count for the depression of LV inotropic and lusitropic functions. However, further studies, in particular, on cardiomyocyte energy metabolism under hypoxic conditions are necessary to draw substantial conclusions on the causes for the observed LV depression.

### Effects of hypoxia, fluid load, and adrenergic blockade on lung injury

Acute hypoxia with elevated fluid load induced pulmonary injury characterized by significant PE and involvement of inflammatory processes. While PE was clearly visible in histologic slices after 6 h of hypoxia, signs of inflammations were sparse at this time. However, mRNA overexpression of inflammatory cytokines such as IL-6 and TNFAIP1 was already present. Several studies have reported that pro-inflammatory cytokines occur at an early stage of hypoxia-induced injury, often in parallel with PE formation [21, 28, 38]. The expression of IL-6 is stimulated by norepinephrine and adrenergic agonists [7, 39]. Our results showed that prolonged hypoxia with fluid infusion upregulated IL-6 expression in the lung. Adrenergic blockade in hypoxia prevented IL-6 overexpression suggesting that sympathetic activation contributes to the inflammatory pulmonary reaction to hypoxia. Hypoxia also stimulates upregulation of TNFα [21, 28]. TNFAIP1 is an endothelial TNFα response factor, thus reflecting increased TNFα secretion [47]. TNFAIP1 expression increased significantly under hypoxic conditions but only in animals with fluid infusion indicating that additional fluid load aggravates pulmonary inflammation. Adrenergic blockade had no effect on TNFAIP1.

It is widely accepted that hypoxia-related PE such as HAPE results from increased pulmonary capillary pressure due to uneven pulmonary vasoconstriction [4, 29]. Hypoxia with increased fluid load promoted PE formation, most likely due to further elevation of pulmonary capillary pressure. This effect was not attenuated by adrenergic blockade, which may be at least partly explained by the reduced amounts of PF. Although PE was mostly milder with fluid injection, it was only slightly mitigated by adrenergic blockade. These results suggest that the effects of hypoxic sympathetic activation have lower impact on PE formation than fluid load, at least in a physiological range of sympathetic activation. Notably, combination of hypoxia and NE infusion as a model of sympathetic overactivation aggravated the severity of PE [6]. In most of the hypoxic animals of the present study, PE was mild to moderate with the alveoli remaining free from fluid. This is shown in lung histology and is confirmed by the W/D ratio. Lung histology showed an interstitial edema with mostly intact alveolo-capillary walls as it is typical for hydrostatic edema. This is in correspondence with the W/D ratios, which were below 6 in all normoxic and hypoxic groups indicating absence of severe alveolar edema [32].

The lung has several mechanisms for counteracting PE formation such as alveolar fluid clearance (AFC) or formation of pleural effusion. Drainage into the pleural cavity mainly serves to prevent alveolar flooding [8, 46]. This is, for example, illustrated in the 6 h NaCl,H-inf group showing a significantly elevated PEI but, in parallel, a significantly increased PF volume (Fig. 2). This protective mechanism may have, at least partially, compensated for the additional fluid influx and might have prevented more severe PE. Remarkably, very low amounts of PF were found in the 1.5 h NaCl,H-inj and 6 h PZ + PR,H-inj groups, which presented particularly high PEI values (Fig. 2A and B) indicating that compensatory mechanisms might have been overridden. A similar result has been found in normoxic rats infused with the α-adrenergic agonist phenylephrine or with NE plus the β-blocker propranolol [36]. AFC, which is mediated by β_2_-adrenergic pathways [31], serves to remove fluid from the alveoli. Hypoxia impairs β_2_-adrenergic signaling in the alveolar epithelium and reduces AFC [25]. If PE is moderate and primarily confined to the interstitium, impaired AFC has no major consequences. Accordingly, β-adrenergic blockade in hypoxia had no marked effect on PEI. However, pro- and anti-edematous mechanisms form a very sensitive balance. Abrupt and strong adrenergic blockade with injection of PZ + PR may accelerate and aggravate development of lung injury and impedes compensatory mechanisms. If this occurs in association with prolonged hypoxia and compromised LV function, severe PE might result such as in the 6 h PZ + PR,H-inj group.

### Cardiopulmonary interactions in hypoxia

The compromised LV inotropic function may result in a backlog into the pulmonary circulation as can be inferred from the impaired LV relaxation (LV dP/dt min) and the higher PEI in the groups with 6 h of hypoxia and fluid infusion. In hypoxia, pulmonary arterial and capillary pressures increase due to hypoxic pulmonary vasoconstriction. This leads to prolongation of RV ejection time [26]. In the 6 h NaCl,H-inf group, LVSP was significantly reduced but RVSP was still on normoxic level meaning a mismatch in the pump function of the two ventricles. This imbalance between the function of RV and LV may have contributed to the high PEI in these animals.

Another case of RV-LV imbalance is given in the 1.5 h NaCl,H-inj group. These animals presented a markedly elevated RVSP, while LVSP was slightly below normoxic level. This observation is in full accordance with a study on mice showing that acute hypoxia induced a divergence between LV and RV systolic function with a significant increase in RVSP [9]. In the 1.5 h NaCl,H-inj group, PEI was significantly increased compared to normoxic animals and even to the 1.5 h NaCl,H-inf group, while the amount of PF was very low (see Fig. 2A and B). We assume that the RVSP elevation in the 1.5 h NaCl,H-inj group resulted from the rapid injection of a fluid bolus of about 3% of total blood volume. In conclusion, the results underline the high importance of an appropriate fluid management under hypoxic conditions. This applies both to travelers to high altitude and to patients under hypoxic conditions.

### Limitations of the study

To reduce fluid administration, particularly in the final period of the experiment, we administered NaCl or adrenergic blockers as injection. The amount of injected fluid of 0.5 ml is 3% of the average blood volume of the rat [33], which is considered to be a very low fluid volume. However, while infusion ensures a constant and even drug delivery, with injection a drug bolus with an initially high and then gradually declining concentration is administered. Consequently, the effective drug dose at the end of the experiment cannot be estimated with sufficient reliability. On the one hand, administration of drugs by infusion is more beneficial than injection if infusion rate is strictly reduced. On the other hand, infusion of 0.5 ml over a very short interval of time holds a considerable risk to administer an indefinite amount of the drug, whereas injection ensures administration of the correct drug quantity. For studying hypoxic exposure over longer periods of time, correct fluid amounts and drug doses can be ensured with infusion at greatly reduced infusion rate.

Analysis of inflammation markers in the lung at mRNA level might also be considered to be a limitation of this study. Based on our previous findings [38], we had not expected a significant increase in protein concentration of inflammatory cytokines within 6 h of hypoxia. We are aware of the fact that modulation of mRNA expression is not necessarily related to changes in protein concentration, but the observed increase in TNFAIP1 mRNA expression is a clear indication that inflammatory processes contribute to hypoxic lung injury.

We have not investigated the effects of adrenergic blockers under normoxic conditions as this was not the principal focus of this study. Comparison of our results with existing data on the effects of adrenergic blockers on cardiovascular function in normoxic rats (e.g., [20, 49]) indicated differences that are attributable to hypoxia.

Finally, the results of this study cannot explain the reasons for hypoxic depression of LV inotropic and lusitropic functions and its aggravation by increase fluid load. Impaired LV energy metabolism and a contribution of ventricular stretch receptors are two mechanisms supposed to be involved, but further research on causal relations is necessary.

## Conclusions

- Acute hypoxia over 6 h combined with increased fluid load depressed LV inotropic and lusitropic function and induced pulmonary injury characterized by inflammation and edema. Reduced fluid load attenuated these cardiac and pulmonary effects of hypoxia.
- Sympathetic activation in hypoxia was mild and could only partially compensate for hypoxia-induced LV depression. Combination of hypoxia with increased fluid load even compromises sympathetic activation or sympathetic effects on the heart. Ventricular stretch reflexes or hypoxic impairment of myocardial energy metabolism might be possible explanations, but further studies are necessary.
- Factors increasing pulmonary perfusion or pulmonary capillary pressure promote PE formation. Sympathetic activation plays a minor role for PE formation in acute hypoxia. However, even with low fluid load, adrenergic blockade cannot prevent PE. This may be attributed to the differential effects of the various factors on the delicate balance between pro- and anti-edematous mechanisms.
- Under acute hypoxic conditions, appropriate fluid management has particular importance for adequate cardiopulmonary function.

## Data Availability

All data generated or analyzed during this study are included in this published article. The research has been performed in animals. All animal protocols were approved by the Federal State Agency. The experiments were conducted in accordance with the Guide for the Care and Use of Laboratory Animals published by the National Institutes of Health and with the “European Convention for the Protection of Vertebrate Animals used for Experimental and other Scientific Purposes” (Council of Europe No 123, Strasbourg 1986).
